# Potential of Serum ApoC1 as a Noninvasive Biomarker for Breast Cancer Detection and Prognosis: A Prospective Case–Control Study

**DOI:** 10.1002/cnr2.70359

**Published:** 2025-10-10

**Authors:** Abolfazl Khalafi‐Nezhad, Mahdi Barazesh, Ahmad Abdollahi, Negin Kheiri, Marzieh Amani

**Affiliations:** ^1^ Hematology Research Center, Department of Hematology, Medical Oncology and Stem Cell Transplantation Shiraz University of Medical Sciences Shiraz Iran; ^2^ Cellular and Molecular Research Center Gerash University of Medical Sciences Gerash Iran; ^3^ Department of Oncosurgery Gerash University of Medical Sciences Gerash Iran; ^4^ Department of Internal Medicine Shiraz University of Medical Sciences Shiraz Iran

**Keywords:** apolipoprotein C1, breast cancer, diagnostic performance, serum biomarker

## Abstract

**Background and Aims:**

Breast cancer (BC) is one of the most frequent malignancies. Apolipoprotein C1 (ApoC1) has been known as a promising therapeutic target and valuable prognostic and diagnostic biomarker in cancers. The aim of this study was to explore the diagnostic implication of serum ApoC1 concentration in new cases of BC.

**Methods:**

This prospective case–control study was conducted on 76 new cases of BC patients referred to Amir‐al‐Momenin Gerash hospital. Subjects in the control group were 15 healthy individuals. The serum concentrations of ApoC1 of the control group and the BC patients were measured by ELISA assay. Data analysis was performed using Student's *t*‐test and one‐way analysis of variance (ANOVA) followed by Dunnett's post hoc test.

**Results:**

The mean age of the BC patients was 49.21 ± 10.42 years. In terms of diagnostic performance, ApoC1 concentration showed a significant down‐regulation in female BC patients compared to healthy controls. The receiver operating characteristic (ROC) curve analysis indicated an area under the curve (AUC) of 1.00, demonstrating the excellent diagnostic value of ApoC1 in distinguishing BC patients from healthy individuals. The optimal cutoff value for ApoC1 concentration was determined to be 15.4 mg/dL, with 100% sensitivity, 100% specificity, and 100% accuracy. Furthermore, the prognostic analysis focused on HER2 over‐expression patients demonstrated a statistically significant difference in ApoC1 concentration compared to other types of BC. The ROC curve analysis revealed the prognostic performance of ApoC1, with an optimal cutoff value of 5.6 mg/dL, 100% positive predictive value (PPV), and 100% negative predictive value (NPV).

**Conclusion:**

According to the findings of the current study, ApoC1 may be a potential serum biomarker for the diagnosis of BC. Moreover, ApoC1 can be considered a highly accurate prognostic biomarker for BC.

## 1 Introduction

One of the most common cancers among women in the United States is breast cancer (BC) [1]. Moreover, BC is also among the most frequently diagnosed malignancies in Iranian women. While there have been some studies exploring the epidemiological aspects of BC among Iranian women, current knowledge remains incomplete, and further research is needed to better understand the disease's prevalence and characteristics in this population. The occurrence rate of BC among females is estimated to be 22 cases per 100 000 individuals [2].

According to studies, early detection of BC combined with appropriate therapy can significantly reduce long‐term mortality [3, 4, 5]. Mammography is the standard screening method for breast cancer; however, it is less effective for women under 40 years of age. This technique is also less sensitive in detecting small tumors and is limited in patients with dense breast tissue. Furthermore, it does not provide prognostic information regarding disease outcomes [6, 7]. Magnetic resonance imaging (MRI) can detect small lesions that may be missed by mammography; however, MRI is less specific and more prone to false‐positive results, which can lead to overdiagnosis and unnecessary interventions [8, 9]. None of these methods—including ultrasound, CT scan, PET scan, or existing serum markers—are considered entirely reliable for screening purposes [1].

The discovery of novel biomarkers is a growing focus in cancer research, offering clinicians new tools for predicting recurrence, survival, and treatment response [10]. A significant drawback of many existing tumor biomarkers is the requirement for biopsy or surgical tissue samples. Consequently, the use of biomarkers detectable in easily accessible body fluids such as blood, saliva, or urine is highly desirable, as they offer noninvasive, repeatable methods for monitoring disease progression and therapeutic response [11].

Apolipoproteins (Apos) are a group of proteins involved in lipid transport as components of lipoproteins. In humans, 22 Apo members are categorized into 11 subgroups: ApoA, ApoB, ApoC, ApoD, ApoE, ApoF, ApoH, ApoL, ApoM, ApoO, and ApoJ [12]. Apos have emerged as potential biomarkers for both diagnosis and prognosis in various cancers, including lung, gastric, and colorectal cancers [13, 14]. These proteins have been implicated in key oncogenic pathways such as PI3K/Akt, MAPK, and Wnt signaling [15, 16]. Apos contribute to cancer progression by regulating critical mechanisms, including resistance to apoptosis, promotion of inflammation and oxidative stress, drug resistance, angiogenesis, metastasis, and sustained proliferation [17]. Given their biological roles, some Apos have also been explored as potential therapeutic targets [18].

Among these, apolipoprotein C (ApoC) has shown a complex and somewhat contradictory role in cancer biology. The ApoC family consists primarily of ApoCI, ApoCII, and ApoCIII, which are predominantly synthesized in the liver and, to a lesser extent, in the intestine. While ApoCI and ApoCII are found in all lipoproteins except LDL, ApoCIII is also present on the LDL surface [19]. Recent findings suggest ApoC is not only a promising therapeutic target but also a valuable prognostic and diagnostic biomarker in multiple malignancies [16]. For example, Takano et al. [20] reported that elevated tissue and serum ApoC1 levels were associated with poor prognosis in pancreatic cancer patients.

Other studies have demonstrated anti‐cancer effects of purified ApoC1 peptides in breast cancer models. These peptides inhibited the proliferation of BC cells in vitro and suppressed tumor growth in xenograft models using athymic nude mice [21]. Moreover, overexpression of ApoC1 has been shown to distinguish triple‐negative breast cancer (TNBC) from non‐TNBC and is considered a potential prognostic marker for TNBC [22]. In prostate cancer, upregulation of ApoC1 mRNA and protein expression supports cell survival by inhibiting apoptosis. Higher serum ApoC1 levels have also been correlated with poor prognosis in prostate cancer patients [23].

Conversely, reduced ApoC1 levels have been observed in the serum of patients with non‐small cell lung cancer (NSCLC), suggesting a possible tumor‐suppressive role in this context [24]. In papillary thyroid carcinoma (PTC), downregulation of ApoC1 was associated with higher clinical stages. A ProteinChip‐array‐based immunoassay used to analyze crude serum samples confirmed a gradual decrease in ApoC1 levels with advancing PTC [25].

Given the high incidence of breast cancer and the conflicting data surrounding the role of ApoC1 in cancer progression and prognosis, particularly in the absence of comprehensive studies within our country, this study aimed to assess the diagnostic value of serum ApoC1 in newly diagnosed cases of breast cancer.

## 2 Methods

### 2.1 Ethical Consideration

The current study was approved by the Ethical Committee of Shiraz University of Medical Sciences (IR.SUMS.MED.REC.1400.617). Written informed consent was obtained from all participants prior to enrollment. Moreover, the study protocols were conducted in accordance with the ethical standards outlined in the 1964 Declaration of Helsinki and its subsequent amendments.

### 2.2 Sample Selection

This prospective case–control study was conducted on 76 Iranian patients with BC who were referred to Amir‐al‐Momenin Gerash Hospital from April to December 2022.

The control group consisted of 15 healthy individuals who were carefully matched to the BC group based on several demographic characteristics. Specifically, the control participants were matched for age, menstrual status, marital status, and body mass index (BMI). Furthermore, all control individuals had no history of cancer, chronic disease, or active inflammatory conditions. This rigorous matching approach was used to reduce potential confounding variables and increase the internal validity of the study. All participants were enrolled voluntarily and incurred no cost for participation.

### 2.3 Sample Size Calculation

The sample size was calculated according to Equation (1):

(1) $$n=\frac{Z_{1-\frac{\alpha }{2}}^{2}\times p\left(1-p\right)}{d^{2}}$$

In this study, the power was set to 80%, and *α* = 0.05; therefore, the sample size included 76 patients with BC and 15 healthy individuals.

### 2.4 Procedure

Patient clinical data, including age, tumor size, nuclear grade, venous and lymphatic involvement, lymph node metastasis, estrogen receptor (ER), progesterone receptor (PR), and human epidermal growth factor receptor 2 (HER2), was extracted from medical records.

Tumor classification was based on the latest edition of the AJCC and the TNM Classification system [26].

### 2.5 Inclusion and Exclusion Criteria

Patients with a definitive diagnosis of BC were included. Patients who had previously undergone chemotherapy or radiotherapy, as well as those with acute or chronic underlying conditions (e.g., diabetes, hyperlipidemia, infections, kidney disease, rheumatic diseases, other malignancies) were excluded.

### 2.6 ApoC1 Assay

Blood samples were collected from all participants. Serum was separated and stored at −80°C. Serum ApoC1 concentration was measured using a sandwich ELISA kit (Elabscience Biotechnology Co. Ltd.) following the manufacturer's protocol. To enhance reliability, all tests were performed in duplicate.

### 2.7 Statistical Analysis

Data were presented as mean ± standard deviation (SD) or frequency (percentage). The Shapiro–Wilk test was used to assess normality. This test evaluates the null hypothesis that the data are normally distributed. A *p*‐value > 0.05 indicates normal distribution [27].

To assess the equality of variances, Levene's test was applied. A *p*‐value > 0.05 indicates homogeneity of variances across groups [28]. Student's *t*‐test and one‐way ANOVA with Dunnett's post hoc test were used for two‐group and multi‐group comparisons, respectively.

Logistic regression analysis was used to explore associations between ApoC1 concentrations and clinical variables such as age, disease stage, and lymph node involvement. ROC curve analysis was performed to evaluate the diagnostic capability of ApoC1. Data were analyzed using SPSS software (version 16.0, SPSS Inc., Chicago, IL), and MedCalc software (version 20.1.4) was used for ROC analysis. A *p*‐value < 0.05 was considered statistically significant.

## 3 Results

### 3.1 Baseline Characteristics of Female Breast Cancer Patients

This study included 76 female BC patients and 15 healthy controls. The mean ± SD age and BMI of the BC group were 49.21 ± 10.42 years (range: 26–75 years) and 25.84 ± 4.75 kg/m^2^ (range: 13.6–34.6 kg/m^2^), respectively. In the control group, the mean ± SD age and BMI were 45.13 ± 12.1 years (range: 25–65 years) and 23.46 ± 4.76 kg/m^2^ (range: 15.2–32.4 kg/m^2^), respectively.

Most BC patients did not have a family history of cancer (86.8%, *n* = 66) and were either overweight (35.5%, *n* = 27) or obese (17.1%, *n* = 13). Additionally, 90.8% of the women were married, and 94.3% had at least one child. The number of children ranged from 0 to 11, with a median of 3.

Patients were categorized into the following BC subtypes: luminal A (51.3%, *n* = 39), luminal B (17.1%, *n* = 13), basal type (13.2%, *n* = 10), and HER2 overexpression (18.4%, *n* = 14).

Disease stages were classified as follows: Stage I (23.7%, *n* = 18), Stage II (46%, *n* = 35), Stage III (25%, *n* = 19), and Stage IV (5.3%, *n* = 4). Additional baseline and clinicopathological characteristics are shown in Table 1.

**TABLE 1:** Demographic and basic clinicopathological details of female BC patients in the study (*N* = 76).

Variables	Category	*N* (%)	Mean ± SD of ApoC1	*p*
Age categories, years	≤ 40	17 (22.4)	9.35 ± 4.01	0.237
	> 40	59 (77.6)	10.48 ± 3.27	
Time from diagnostic, years	≤ 2.5	41 (53.9)	9.90 ± 3.72	0.376
	> 2.5	35 (46.1)	10.60 ± 3.12	
Family history of BC	Yes	10 (13.2)	8.64 ± 3.93	0.121
	No	66 (86.8)	10.46 ± 3.34	
Menopausal status	Pre	43 (56.6)	10.19 ± 3.35	0.924
	Post	33 (43.4)	10.27 ± 3.63	
Marital status	Married (including divorced/widowed)	69 (90.8)	10.29 ± 3.40	0.619
	Unmarried	7 (9.2)	9.60 ± 4.12	
Body max index	Underweight	6 (7.1)	10.48 ± 3.36	0.714
	Normal	30 (35.6)	10.08 ± 3.55	
	Overweight	27 (35.5)	10.72 ± 3.12	
	Obese	13 (17.1)	9.40 ± 4.09	
Number of children	≤ 3	48 (63.1)	10.07 ± 3.51	0.857
	> 3	28 (36.9)	10.22 ± 3.47	
Type of diseases	Early stage	36 (47.4)	10.55 ± 3.28	0.720
	Locally advance	30 (39.5)	9.85 ± 3.62	
	Metastatic	10 (13.2)	10.62 ± 3.78	
TNM stage	I	18 (23.7)	10.41 ± 3.50	0.853
	II	35 (46.1)	9.89 ± 3.91	
	III	19 (25.0)	10.44 ± 2.90	
	IV	4 (5.3)	11.26 ± 1.32	
Histopathological grade at diagnosis	I	9 (11.8)	9.78 ± 3.86	0.401
	II	49 (64.5)	10.62 ± 3.32	
	III	18 (23.7)	9.38 ± 3.63	
Subtype	Luminal A	39 (51.3)	11.64 ± 1.97	< 0.001
	Luminal B	13 (17.1)	11.63 ± 0.99	
	Basal type	10 (13.2)	11.79 ± 1.99	
	HER2 over‐expression	14 (18.4)	3.87 ± 0.63	
Axillary lymph nodes status	Positive	43 (56.6)	10.44 ± 3.28	0.542
	Negative	33 (43.4)	9.95 ± 3.70	
Lymph vascular invasion status	Positive	27 (35.5)	10.50 ± 3.10	0.605
	Negative	49 (64.5)	10.07 ± 3.65	
Estrogen receptor‐status	Positive	50 (65.8)	11.76 ± 1.51	< 0.001
	Negative	26 (34.2)	7.28 ± 4.19	
Progesterone receptor‐status	Positive	40 (52.6)	11.96 ± 1.53	< 0.001
	Negative	36 (47.4)	8.29 ± 3.95	
HER2 receptor‐status	Positive	14 (18.4)	3.87 ± 0.63	< 0.001
	Negative	62 (81.6)	11.66 ± 1.79	

### 3.2 Association Between ApoC1 and Baseline Variables

Associations between serum ApoC1 concentration and demographic/clinical data were analyzed using *t*‐tests and ANOVA. Results are summarized in Table 1.

### 3.3 Diagnostic Performance of ApoC1

**FIGURE 1:**
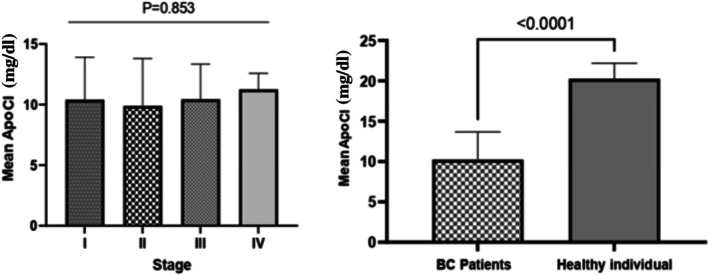
Comparison of mean (±SD) serum ApoC1 concentrations among breast cancer patients stratified by disease stage (Stages I–IV), with healthy controls included for reference. This figure illustrates the progressive decline in ApoC1 levels across advancing stages of breast cancer compared to the control group.

To evaluate diagnostic performance, ApoC1 levels were compared between 76 BC patients and 15 healthy controls. A significant difference in mean ApoC1 levels was found between the groups (Table 2, Figure 1). The effect size (ES = 3.05) indicated a strong clinical effect.

**TABLE 2:** The comparison of the different groups (the case and control groups, and Her2 over‐expression).

Variable			*p*	Effect size
Group	Case (*n* = 76)	Control (*n* = 15)		
ApoCI	10.22 ± 3.45	20.25 ± 1.91	< 0.0001	3.05

Group	Her2 cases (*n* = 14)	Other patients (*n* = 62)	*p*	Effect size
ApoCI	3.87 ± 0.63	11.66 ± 1.79	< 0.0001	4.73

*Note:* Data were presented as mean ± SD. Hedges' *g* formula was used for effect size. Other patients including Luminal A, Luminal B, and Basal type.

**FIGURE 2:**
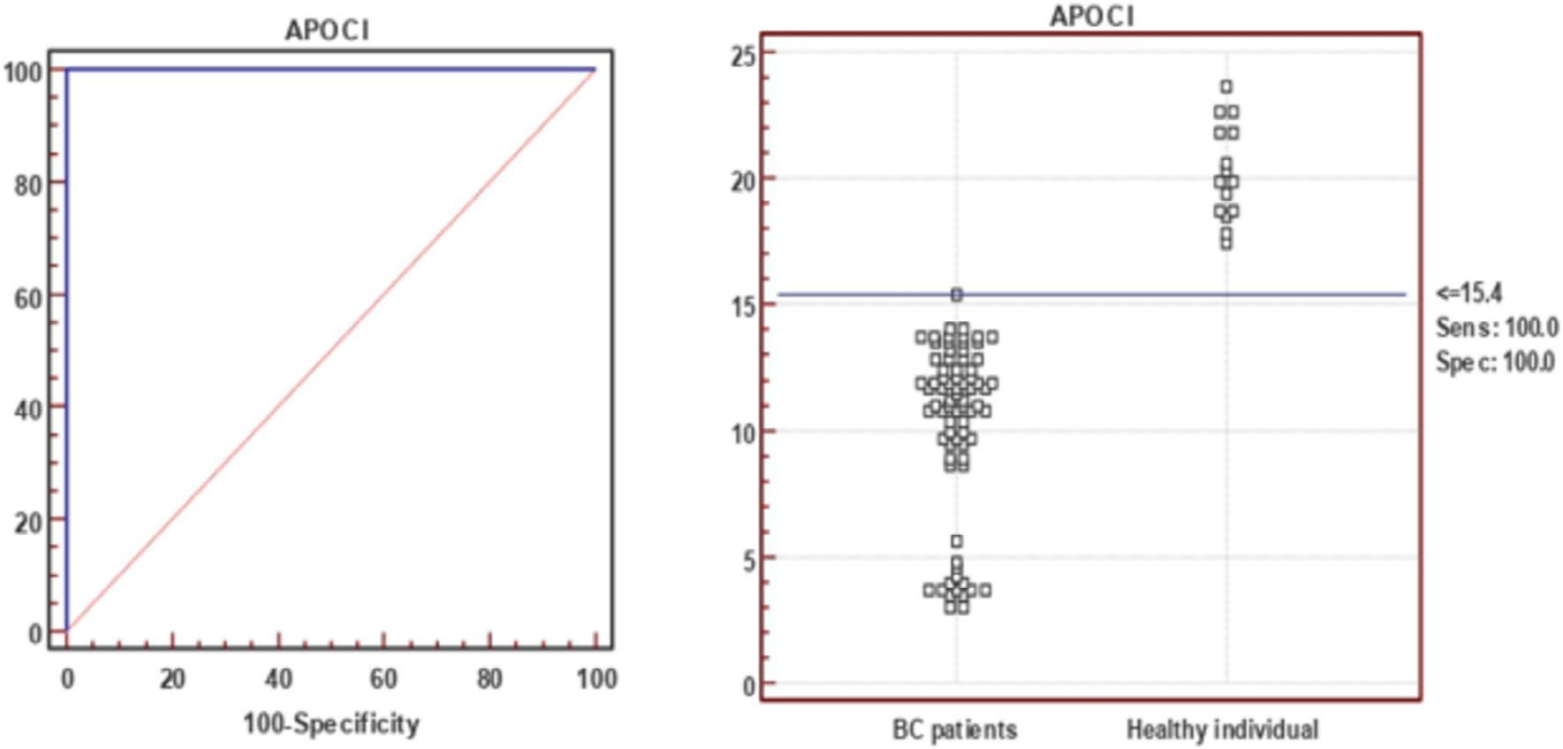
ROC curve demonstrating the diagnostic performance of ApoC1 for differentiating breast cancer patients from healthy individuals.

ApoC1 was significantly downregulated in BC patients (*p* < 0.001). ROC analysis showed an AUC of 1.00 (95% CI, 0.96–1.00; *p* < 0.001), demonstrating excellent diagnostic performance (Figure 2, Table 3).

**TABLE 3:** Analysis of ROC curve to evaluate the diagnostic performance and prognosis of ApoC1.

Index	Value (95% CI)	
	Diagnostic	Prognostic
AUC	1.0 (0.96–1.0)	0.99 (0.99–1.0)
Sensitivity	100 (95.2–100)	92.9 (66.13–99.82)
Specificity	100 (78.0–100)	98.39 (91.34–99.96)
Positive predictive value	100 (100–100)	92.86 (64.92–98.92)
Negative predictive value	100 (100–100)	98.39 (90.22–99.75)
Accuracy	100 (96–100)	97.37 (90.82–99.68)

*Note:* It is more appropriate to use predictive value in diagnostic/prognostic tests while sensitivity–specificity in screening tests.

K‐fold cross‐validation (*k* = 10) was performed to ensure model robustness and reduce overfitting. The dataset was divided into 10 equal folds, and each fold served once as the validation set. Performance metrics from all iterations were averaged to validate the model's generalizability.

At the optimal cutoff of 15.4 mg/dL, ApoC1 achieved 100% sensitivity, specificity, PPV, and NPV, with an overall accuracy of 100% (Figure 2).

### 3.4 Prognostic Performance of ApoC1

**FIGURE 3:**
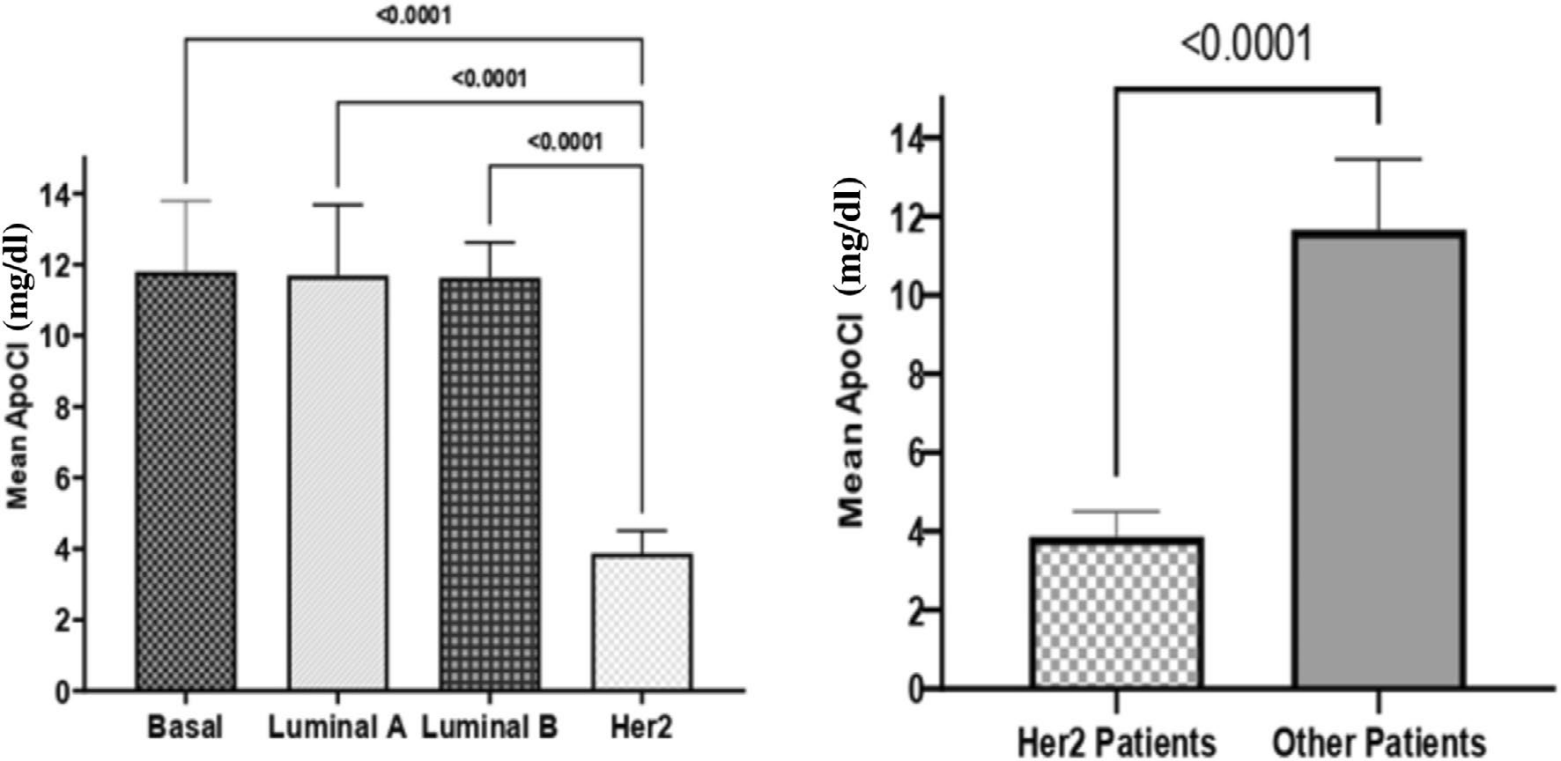
Comparison of serum ApoC1 levels between HER2‐positive breast cancer patients and other molecular subtypes, including basal‐like, luminal A, and luminal B. This figure highlights the significantly lower ApoC1 expression observed in the HER2+ group compared to the other subtypes.

To assess prognostic value, patients were stratified based on HER2 expression. ANOVA revealed a significant difference in ApoC1 levels between the HER2+ group and each of the other subtypes (*p* < 0.0001 for all comparisons) (Figure 3). A *t*‐test confirmed that HER2+ patients had significantly lower ApoC1 levels compared to non‐HER2 patients (*p* < 0.0001) (Table 2, Figure 3).

**FIGURE 4:**
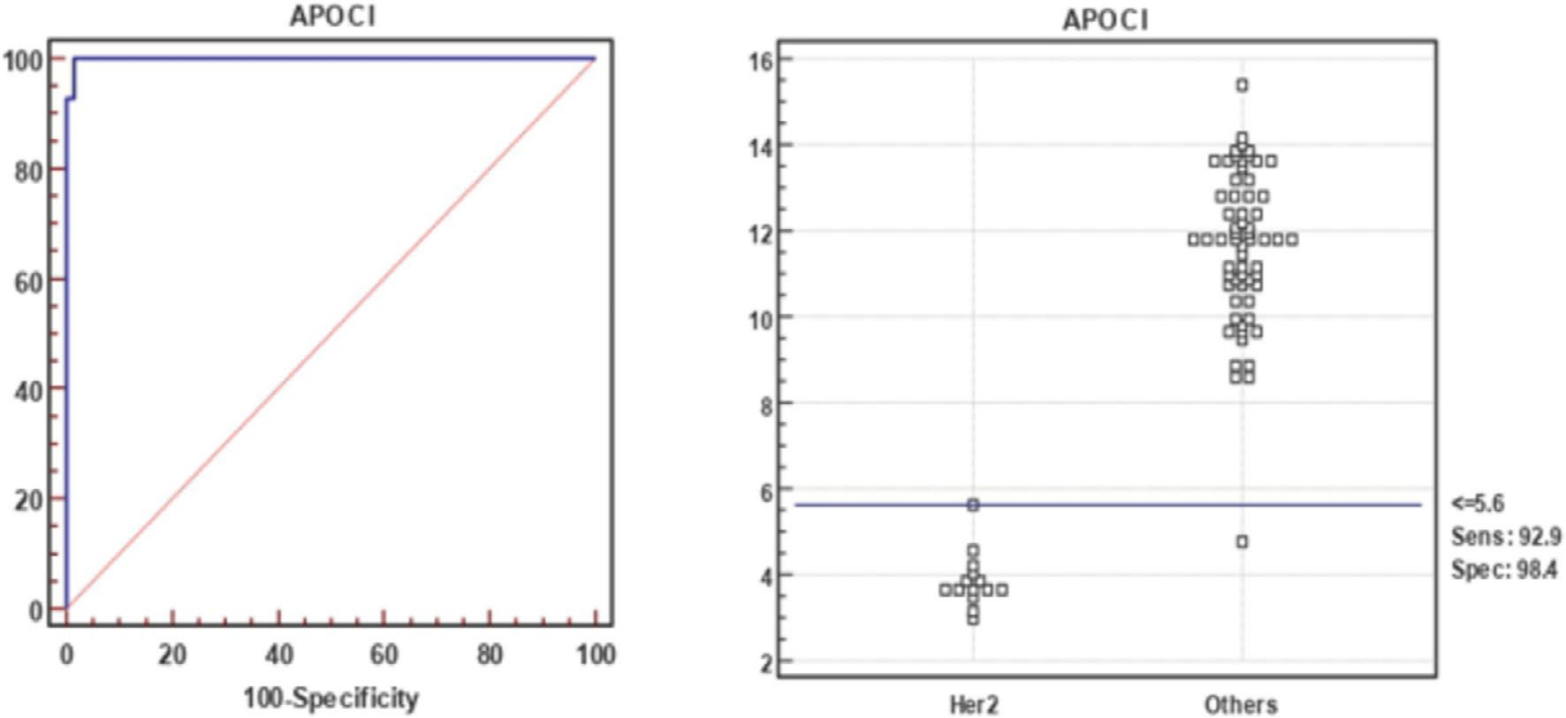
ROC curve illustrating the diagnostic efficacy of ApoC1 for identifying HER2+ patients compared to other breast cancer groups.

ROC analysis (Figure 4, Table 3) revealed that an ApoC1 cutoff of 5.6 mg/dL distinguished HER2+ patients with 100% PPV and NPV. All HER2+ patients were below this threshold except for one luminal A patient (57 years old) whose ApoC1 level was 4.76 mg/dL.

## 4 Discussion

This study aimed to investigate the diagnostic potential of serum Apolipoprotein C1 (ApoC1) in newly diagnosed breast cancer (BC) patients. Our results demonstrated that serum ApoC1 levels could differentiate BC patients from healthy controls with 100% sensitivity, specificity, and predictive values. Notably, ApoC1 also showed robust performance in distinguishing HER2‐positive subtypes, suggesting its dual diagnostic and subtype‐predictive capabilities.

Early diagnosis of breast cancer remains a cornerstone in improving survival outcomes. While conventional imaging methods like mammography and MRI have contributed to early detection, they often fall short due to limitations related to cost, access, radiation exposure, and reduced sensitivity in younger women [1, 29]. These limitations highlight the need for accurate, minimally invasive, and cost‐effective biomarkers that can supplement or even replace current strategies.

Serum, as a non‐invasive and widely accessible biological sample, holds promise for biomarker discovery. Proteomic studies have explored numerous candidates for BC diagnosis, but reproducibility and standardization issues have hindered clinical translation [30, 31, 32]. Within this context, apolipoproteins (Apos) have gained traction due to their biological relevance and stable detectability in serum [33, 34].

ApoC1 is a small (6.6 kDa) protein predominantly expressed in the liver and involved in lipid metabolism, immune regulation, and cellular signaling [13, 34, 35, 36]. It has been implicated in several diseases, including neurodegenerative disorders and malignancies such as pancreatic, lung, and colorectal cancers [20, 37, 38, 39, 40, 41, 42]. In BC, its role is still evolving. Earlier reports using SELDI‐TOF and MALDI‐TOF–MS techniques showed decreased serum ApoC1 in BC patients and identified anti‐tumor activity through peptide characterization, particularly in triple‐negative breast cancer (TNBC) models [21, 22, 43].

Our study corroborates these findings, reporting significantly reduced ApoC1 levels in the BC cohort compared to healthy individuals. Furthermore, lower ApoC1 levels were associated with HER2 overexpression, aligning with prior studies that associate HER2 positivity with worse prognosis [44, 45, 46]. Since HER2 signaling is linked to altered lipid metabolism and apolipoprotein regulation, these metabolic shifts may explain the observed inverse relationship [47]. Thus, ApoC1 may represent a metabolic link between lipid regulation and oncogenic signaling in HER2‐driven BC.

Beyond breast cancer, ApoC1 has shown diagnostic and prognostic utility in various cancers, including colorectal, pancreatic, and renal malignancies [20, 42, 48, 49, 50, 51, 52, 53]. In acute myeloid leukemia, ApoC1 has been shown to influence histone modification and support leukemic proliferation, further underscoring its pleiotropic oncogenic potential [54, 55]. These findings suggest that ApoC1 may function not only as a disease marker but also as a potential therapeutic target across tumor types.

In this study, we identified a diagnostic cutoff of 15.4 mg/dL for ApoC1, which yielded perfect predictive metrics in distinguishing BC from controls. This excellent performance underscores ApoC1's promise as a reliable biomarker. However, variations in reported ApoC1 levels across studies [15, 56] may arise from differing sample preparation, population heterogeneity, or analytical platforms. These discrepancies reinforce the need for standardized protocols and external validation before clinical implementation.

The strengths of this study include the use of rigorous statistical methodology, standardized ELISA‐based assays, and an independent external validation cohort. These elements collectively enhance the reproducibility and reliability of our findings. Additionally, the study design allowed exploration of subtype‐specific differences in ApoC1 levels, reinforcing its clinical relevance.

Nonetheless, several limitations must be acknowledged. The relatively small sample size and limited control group constrain the statistical power and generalizability of our results. Furthermore, clinical parameters such as tumor stage, lymph node status, and hormone receptor expression were not correlated with ApoC1 levels. The age distribution was also narrow, potentially limiting extrapolation to broader age groups. Future studies with larger, more diverse populations are warranted to confirm these findings and explore longitudinal changes in ApoC1 during treatment and follow‐up.

While ELISA is a practical and scalable platform, implementation in routine screening requires consideration of cost, assay standardization, and integration into clinical workflows. Importantly, survival analyses and prognostic modeling are needed to determine whether ApoC1 levels correlate with outcomes such as recurrence and mortality.

## 5 Conclusion

In conclusion, our study highlights the diagnostic and subtype‐discriminating potential of serum ApoC1 in breast cancer. Its high accuracy, coupled with non‐invasive testing, suggests a promising role for ApoC1 as a biomarker in early detection strategies and personalized care, particularly in HER2‐positive subtypes. Continued research to validate and elucidate the biological underpinnings of ApoC1 in BC is essential for translating these findings into clinical practice.

## Author Contributions

**Abolfazl Khalafi‐Nezhad:** conceptualization, supervision, project administration, writing – original draft preparation. **Mahdi Barazesh:** methodology, writing original draft, writing review and editing investigation. **Ahmad Abdollahi:** conceptualization, methodology, writing the original draft, validation. **Negin Kheiri:** data curation, writing – original draft preparation, formal analysis. **Marzieh Amani:** data curation, writing – original draft preparation.

## Ethics Statement

The current study was approved by the Ethical Committee of Shiraz University of Medical Sciences (IR.SUMS.MED.REC.1400.617). The written consent form was signed by all the patients before starting the study. Moreover, the study protocols have been performed according to the ethical standards laid down in the 1964 Declaration of Helsinki and its later amendments.

## Conflicts of Interest

The authors declare no conflicts of interest.

## Data Availability

The data that support the findings of this study are available on request from the corresponding author. The data are not publicly available due to privacy or ethical restrictions.

## References

[1] L. Wang , “Early Diagnosis of Breast Cancer,” Sensors 17, no. 7 (2017): 1572. 28678153 10.3390/s17071572 PMC5539491

[2] N. Nafissi , M. Khayamzadeh , Z. Zeinali , D. Pazooki , M. Hosseini , and M. E. Akbari , “Epidemiology and Histopathology of Breast Cancer in Iran Versus Other Middle Eastern Countries,” Middle East Journal of Cancer 9, no. 3 (2018): 243–251.

[3] A. Migowski , “Early Detection of Breast Cancer and the Interpretation of Results of Survival Studies,” Ciência & Saúde Coletiva 20, no. 4 (2015): 1309. 25923642 10.1590/1413-81232015204.17772014

[4] R. Sheikhpour , M. Agha Sarram , M. R. Zare Mirakabad , and R. Sheikhpour , “Breast Cancer Detection Using Two‐Step Reduction of Features Extracted From Fine Needle Aspirate and Data Mining Algorithms,” Iranian Journal of Breast Diseases 7, no. 4 (2015): 43–51.

[5] Y. Wang , Z. Zhang , X. Mi , et al., “Elevation of Effective p53 Expression Sensitizes Wild‐Type p53 Breast Cancer Cells to CDK7 Inhibitor THZ1,” Cell Communication and Signaling 20, no. 1 (2022): 96. 36058938 10.1186/s12964-022-00837-z PMC9442925

[6] B. N. Hellquist , K. Czene , A. Hjälm , L. Nyström , and H. Jonsson , “Effectiveness of Population‐Based Service Screening With Mammography for Women Ages 40 to 49 Years With a High or Low Risk of Breast Cancer: Socioeconomic Status, Parity, and Age at Birth of First Child,” Cancer 121, no. 2 (2015): 251–258. 25242087 10.1002/cncr.29011

[7] T. Onega , L. E. Goldman , R. L. Walker , et al., “Facility Mammography Volume in Relation to Breast Cancer Screening Outcomes,” Journal of Medical Screening 23, no. 1 (2016): 31–37. 26265482 10.1177/0969141315595254

[8] D. Roganovic , D. Djilas , S. Vujnovic , D. Pavic , and D. Stojanov , “Breast MRI, Digital Mammography and Breast Tomosynthesis: Comparison of Three Methods for Early Detection of Breast Cancer,” Bosnian Journal of Basic Medical Sciences 15, no. 4 (2015): 64–68. 10.17305/bjbms.2015.616 PMC4690445 26614855

[9] A. M. Hassan and M. El‐Shenawee , “Review of Electromagnetic Techniques for Breast Cancer Detection,” IEEE Reviews in Biomedical Engineering 4 (2011): 103–118. 22273794 10.1109/RBME.2011.2169780

[10] J. Decock , N. Obermajer , S. Vozelj , W. Hendrickx , R. Paridaens , and J. Kos , “Cathepsin B, Cathepsin H, Cathepsin X and Cystatin C in Sera of Patients With Early‐Stage and Inflammatory Breast Cancer,” International Journal of Biological Markers 23, no. 3 (2008): 161–168. 18949742 10.1177/172460080802300305

[11] A. M. DeLouize , G. Eick , S. D. Karam , and J. J. Snodgrass , “Current and Future Applications of Biomarkers in Samples Collected Through Minimally Invasive Methods for Cancer Medicine and Population‐Based Research,” American Journal of Human Biology 34, no. 11 (2022): e23665. 34374148 10.1002/ajhb.23665 PMC9894104

[12] A. Mehta and M. D. Shapiro , “Apolipoproteins in Vascular Biology and Atherosclerotic Disease,” Nature Reviews Cardiology 19 (2021): 168–179. 34625741 10.1038/s41569-021-00613-5

[13] J. Yi , L. Ren , J. Wu , et al., “Apolipoprotein C1 (APOC1) as a Novel Diagnostic and Prognostic Biomarker for Gastric Cancer,” Annals of Translational Medicine 7, no. 16 (2019): 380. 31555694 10.21037/atm.2019.07.59 PMC6736826

[14] Y. Zhang and L. Zheng , “Apolipoprotein: Prospective Biomarkers in Digestive Tract Cancer,” Translational Cancer Research 9, no. 5 (2020): 3712–3720. 35117733 10.21037/tcr-19-2106 PMC8799137

[15] H. Zhang , Y. Wang , C. Liu , et al., “The Apolipoprotein C1 Is Involved in Breast Cancer Progression via EMT and MAPK/JNK Pathway,” Pathology, Research and Practice 229 (2022): 153746. 34952429 10.1016/j.prp.2021.153746

[16] L. Ren , J. Yi , W. Li , et al., “Apolipoproteins and Cancer,” Cancer Medicine 8, no. 16 (2019): 7032–7043. 31573738 10.1002/cam4.2587 PMC6853823

[17] C. Wang , Z. Yang , E. Xu , et al., “Apolipoprotein C‐II Induces EMT to Promote Gastric Cancer Peritoneal Metastasis via PI3K/AKT/mTOR Pathway,” Clinical and Translational Medicine 11, no. 8 (2021): e522. 34459127 10.1002/ctm2.522 PMC8351524

[18] S. C. Delk , A. Chattopadhyay , J. C. Escola‐Gil , A. M. Fogelman , and S. T. Reddy , “Apolipoprotein Mimetics in Cancer,” Seminars in Cancer Biology 73 (2021): 158–168. 33188891 10.1016/j.semcancer.2020.11.002 PMC8110614

[19] S. T. Nielsen , R. M. Lytsen , N. Strandkjær , et al., “Significance of Lipids, Lipoproteins, and Apolipoproteins During the First 14–16 Months of Life,” European Heart Journal 44 (2023): 4408–4418. 37632410 10.1093/eurheartj/ehad547 PMC10635670

[20] S. Takano , H. Yoshitomi , A. Togawa , et al., “Apolipoprotein C‐1 Maintains Cell Survival by Preventing From Apoptosis in Pancreatic Cancer Cells,” Oncogene 27, no. 20 (2008): 2810–2822. 18037960 10.1038/sj.onc.1210951

[21] Y. Sun , J. Zhang , F. Guo , et al., “Identification of Apolipoprotein C‐I Peptides as a Potential Biomarker and Its Biological Roles in Breast Cancer,” Medical Science Monitor: International Medical Journal of Experimental and Clinical Research 22 (2016): 1152–1160. 27052600 10.12659/MSM.896531 PMC4827518

[22] D. Song , L. Yue , J. Zhang , et al., “Diagnostic and Prognostic Significance of Serum Apolipoprotein C‐I in Triple‐Negative Breast Cancer Based on Mass Spectrometry,” Cancer Biology & Therapy 17, no. 6 (2016): 635–647. 27260686 10.1080/15384047.2016.1156262 PMC4990397

[23] W. P. Su , L. N. Sun , S. L. Yang , et al., “Apolipoprotein C1 Promotes Prostate Cancer Cell Proliferation In Vitro,” Journal of Biochemical and Molecular Toxicology 32, no. 7 (2018): e22158. 29719090 10.1002/jbt.22158 PMC6099310

[24] Y. Yang , S. Zhao , Y. Fan , et al., “Detection and Identification of Potential Biomarkers of Non‐Small Cell Lung Cancer,” Technology in Cancer Research & Treatment 8, no. 6 (2009): 455–465. 19925029 10.1177/153303460900800607

[25] Y. Fan , L. Shi , Q. Liu , et al., “Discovery and Identification of Potential Biomarkers of Papillary Thyroid Carcinoma,” Molecular Cancer 8, no. 1 (2009): 79. 19785722 10.1186/1476-4598-8-79 PMC2761863

[26] G. Cserni , E. Chmielik , B. Cserni , and T. Tot , “The New TNM‐Based Staging of Breast Cancer,” Virchows Archiv 472, no. 5 (2018): 697–703. 29380126 10.1007/s00428-018-2301-9

[27] Z. Hanusz , J. Tarasinska , and W. Zielinski , “Shapiro–Wilk Test With Known Mean,” REVSTAT‐Statistical Journal 14, no. 1 (2016): 89–100.

[28] G. V. Glass , “Testing Homogeneity of Variances,” American Educational Research Journal 3, no. 3 (1966): 187–190.

[29] Z. He , Z. Chen , M. Tan , et al., “A Review on Methods for Diagnosis of Breast Cancer Cells and Tissues,” Cell Proliferation 53, no. 7 (2020): e12822. 32530560 10.1111/cpr.12822 PMC7377933

[30] P. E. Geyer , L. M. Holdt , D. Teupser , and M. Mann , “Revisiting Biomarker Discovery by Plasma Proteomics,” Molecular Systems Biology 13, no. 9 (2017): 942. 28951502 10.15252/msb.20156297 PMC5615924

[31] L. Chung and R. C. Baxter , “Breast Cancer Biomarkers: Proteomic Discovery and Translation to Clinically Relevant Assays,” Expert Review of Proteomics 9 (2012): 599–614. 23256671 10.1586/epr.12.62

[32] R. Hong , H. Sun , D. Li , et al., “A Review of Biosensors for Detecting Tumor Markers in Breast Cancer,” Life 12, no. 3 (2022): 342. 35330093 10.3390/life12030342 PMC8955405

[33] Y. He , J. Chen , Y. Ma , and H. Chen , “Apolipoproteins: New Players in Cancers,” Frontiers in Pharmacology 13 (2022): 1051280. 36506554 10.3389/fphar.2022.1051280 PMC9732396

[34] Y. Cui , C. Miao , C. Hou , Z. Wang , and B. Liu , “Apolipoprotein C1 (APOC1): A Novel Diagnostic and Prognostic Biomarker for Clear Cell Renal Cell Carcinoma,” Frontiers in Oncology 10 (2020): 1436. 32974161 10.3389/fonc.2020.01436 PMC7468425

[35] E. V. Fuior and A. V. Gafencu , “Apolipoprotein C1: Its Pleiotropic Effects in Lipid Metabolism and Beyond,” International Journal of Molecular Sciences 20, no. 23 (2019): 5939. 31779116 10.3390/ijms20235939 PMC6928722

[36] A. Rouland , D. Masson , L. Lagrost , B. Vergès , T. Gautier , and B. Bouillet , “Role of Apolipoprotein C1 in Lipoprotein Metabolism, Atherosclerosis and Diabetes: A Systematic Review,” Cardiovascular Diabetology 21, no. 1 (2022): 272. 36471375 10.1186/s12933-022-01703-5 PMC9724408

[37] P. Bus , L. Pierneef , R. Bor , et al., “Apolipoprotein C‐I Plays a Role in the Pathogenesis of Glomerulosclerosis,” Journal of Pathology 241, no. 5 (2017): 589–599. 27976371 10.1002/path.4859

[38] C. S. Ki , D. L. Na , D. K. Kim , H. J. Kim , and J. W. Kim , “Genetic Association of an Apolipoprotein C‐I (APOC1) Gene Polymorphism With Late‐Onset Alzheimer's Disease,” Neuroscience Letters 319, no. 2 (2002): 75–78. 11825674 10.1016/s0304-3940(01)02559-9

[39] G. J. McKay , D. A. Savage , C. C. Patterson , G. Lewis , A. J. McKnight , and A. P. Maxwell , “Association Analysis of Dyslipidemia‐Related Genes in Diabetic Nephropathy,” PLoS One 8, no. 3 (2013): e58472. 23555584 10.1371/journal.pone.0058472 PMC3608831

[40] H. Zhang , Y. Wang , C. Liu , et al., “The Apolipoprotein C1 Is Involved in Breast Cancer Progression via EMT and MAPK/JNK Pathway,” Pathology, Research and Practice 229 (2021): 153746. 34952429 10.1016/j.prp.2021.153746

[41] H. Ren , Z. Chen , L. Yang , et al., “Apolipoprotein C1 (APOC1) Promotes Tumor Progression via MAPK Signaling Pathways in Colorectal Cancer,” Cancer Management and Research 11 (2019): 4917–4930. 31213910 10.2147/CMAR.S192529 PMC6549782

[42] H. L. Ko , Y. S. Wang , W. L. Fong , M. S. Chi , K. H. Chi , and S. J. Kao , “Apolipoprotein C1 (APOC1) as a Novel Diagnostic and Prognostic Biomarker for Lung Cancer: A Marker Phase I Trial,” Thoracic Cancer 5, no. 6 (2014): 500–508. 26767044 10.1111/1759-7714.12117 PMC4704334

[43] Y. Fan , J. Wang , Y. Yang , et al., “Detection and Identification of Potential Biomarkers of Breast Cancer,” Journal of Cancer Research and Clinical Oncology 136 (2010): 1243–1254. 20237941 10.1007/s00432-010-0775-1 PMC11827925

[44] A. M. Gonzalez‐Angulo , J. K. Litton , K. R. Broglio , et al., “High Risk of Recurrence for Patients With Breast Cancer Who Have Human Epidermal Growth Factor Receptor 2‐Positive, Node‐Negative Tumors 1 Cm or Smaller,” Journal of Clinical Oncology: Official Journal of the American Society of Clinical Oncology 27, no. 34 (2009): 5700–5706. 19884543 10.1200/JCO.2009.23.2025 PMC2792998

[45] S. Chia , B. Norris , C. Speers , et al., “Human Epidermal Growth Factor Receptor 2 Overexpression as a Prognostic Factor in a Large Tissue Microarray Series of Node‐Negative Breast Cancers,” Journal of Clinical Oncology 26, no. 35 (2008): 5697–5704. 19001334 10.1200/JCO.2007.15.8659

[46] G. Curigliano , G. Viale , V. Bagnardi , et al., “Clinical Relevance of HER2 Overexpression/Amplification in Patients With Small Tumor Size and Node‐Negative Breast Cancer,” Journal of Clinical Oncology: Official Journal of the American Society of Clinical Oncology 27, no. 34 (2009): 5693–5699. 19884553 10.1200/JCO.2009.22.0962

[47] J. Baumann , M. Kokabee , J. Wong , R. Balasubramaniyam , Y. Sun , and D. S. Conklin , “Global Metabolite Profiling Analysis of Lipotoxicity in HER2/Neu‐Positive Breast Cancer Cells,” Oncotarget 9, no. 43 (2018): 27133–27150. 29930756 10.18632/oncotarget.25500 PMC6007458

[48] J. Y. Engwegen , A. C. Depla , M. E. Smits , et al., “Detection of Colorectal Cancer by Serum and Tissue Protein Profiling: A Prospective Study in a Population at Risk,” Biomarker Insights 3 (2008): 375–385. 19578519 10.4137/bmi.s790 PMC2688344

[49] Y. Jin , Y. Yang , Y. Su , et al., “Identification a Novel Clinical Biomarker in Early Diagnosis of Human Non‐Small Cell Lung Cancer,” Glycoconjugate Journal 36, no. 1 (2019): 57–68. 30607521 10.1007/s10719-018-09853-z

[50] Q. Zhang , J. Wang , R. Dong , S. Yang , and S. Zheng , “Identification of Novel Serum Biomarkers in Child Nephroblastoma Using Proteomics Technology,” Molecular Biology Reports 38, no. 1 (2011): 631–638. 20369385 10.1007/s11033-010-0149-4

[51] E. W. Klee , O. P. Bondar , M. K. Goodmanson , et al., “Candidate Serum Biomarkers for Prostate Adenocarcinoma Identified by mRNA Differences in Prostate Tissue and Verified With Protein Measurements in Tissue and Blood,” Clinical Chemistry 58, no. 3 (2012): 599–609. 22247499 10.1373/clinchem.2011.171637 PMC3951013

[52] L. Ren , J. Yi , W. Li , et al., “Immunophenotyping Analysis of Renal Cell Carcinoma Identified APOC1 as an Immune‐Related Biomarker and Therapeutic Target,” 2021.

[53] Y.‐l. Li , L.‐w. Wu , L.‐h. Zeng , et al., “ApoC1 Promotes the Metastasis of Clear Cell Renal Cell Carcinoma via Activation of STAT3,” Oncogene 39, no. 39 (2020): 6203–6217. 32826950 10.1038/s41388-020-01428-3

[54] W. Tang , H. Liu , X. Li , et al., “Upregulation of APOC1 Promotes Colorectal Cancer Progression and Serves as a Potential Therapeutic Target Based on Bioinformatics Analysis,” Journal of Oncology 2023 (2023): 2611105. 36908705 10.1155/2023/2611105 PMC9995190

[55] X. Yang , B. Lu , X. Sun , et al., “ANP32A Regulates Histone H3 Acetylation and Promotes Leukemogenesis,” Leukemia 32, no. 7 (2018): 1587–1597. 29467488 10.1038/s41375-018-0010-7

[56] Y. Yan , Y. Zhou , K. Wang , Y. Qiao , L. Zhao , and M. Chen , “Apolipoprotein C1 (APOC1), A Candidate Diagnostic Serum Biomarker for Breast Cancer Identified by Serum Proteomics Study,” Critical Reviews in Eukaryotic Gene Expression 32, no. 4 (2022): 1–9. 10.1615/CritRevEukaryotGeneExpr.2021040967 35695660

